# Metabolomic and morphologic surveillance reveals the impact of lactic acid-treated barley on *in vitro* ruminal fermentation

**DOI:** 10.5713/ab.23.0550

**Published:** 2024-05-07

**Authors:** K E Tian, Dicky Aldian, Masato Yayota

**Affiliations:** 1 The United Graduate School of Agricultural Science, Gifu University, Gifu, 501-1193, Japan; 2 College of Animal Science and Technology, Southwest University, Chongqing, 400715/402460, China; 3 Postdoctoral Workstation of Animal Science, Southwest University, Chongqing, 400715/402460, China; 4 Faculty of Applied Biological Sciences, Gifu University, Gifu, 501-1193, Japan

**Keywords:** Barley, *In vitro* Fermentation, Lactic Acid, Metabolome, Morphology

## Abstract

**Objective:**

Lactic acid (LA) treatment of cereals is known to improve ruminant performance. However, changes in cereal nutrient levels and variations in rumen fermentation remain unclear.

**Methods:**

This study was designed to compare the effects of 5% LA treatment on the trophic and morphological characteristics of barley and to discover the differences in rumen fermentation characteristics and metabolomes between LA-treated and untreated barley.

**Results:**

Compared with those of untreated barley (BA), the dry matter (DM), crude protein (CP), ash and water-soluble carbohydrate contents of barley plants treated with 5% LA for 48 h (BALA) decreased, but the resistant starch (RS) and non-fiber carbohydrate contents increased. Moreover, the amount of proteinaceous matrix in BA decreased in response to LA treatment. During *in vitro* fermentation, BALA had a greater pH but lower dry matter disappearance and ammonia, methane, and short-chain fatty acid levels than BA. The differential metabolites between BA and BALA were clustered into metabolic pathways such as purine metabolism, lysine degradation, and linoleic acid metabolism. Observable differences in ultrastructure between BALA and BA were noted during fermentation.

**Conclusion:**

Lactic treatment altered barley nutrient content, including DM, CP, RS, ash, water-soluble carbohydrates and non-fiber carbohydrates, and affected barley ultrastructure. These variations led to significant and incubation time-dependent changes in the *in vitro* fermentation characteristics and metabolome.

## INTRODUCTION

Barley is the fourth most common crop produced worldwide and serves as the primary energy substrate in intensive ruminant rearing systems in European countries and Canada [1,2]. Compared to other cereals such as corn and sorghum, barley contains greater levels of protein, minerals, and vitamins [3]. However, the highly fermentable nature of barley can easily induce digestive disorders when applied to the diet at a high ratio, particularly in beef fattening industries where the concentrate comprises more than 70% (and even more than 90% in some cases) of the total ration for maximizing beef production. Excessive barley in the diet can drastically reduce the ruminal pH, leading to inflammation throughout the body and impacting ruminant performance, thus hindering gastrointestinal barley utilization [4].

Over the past few decades, numerous efforts have been made to improve barley utilization without negatively impacting ruminant health through processing techniques. Lactic acid (LA) has long been used to improve starch productivity in food science [5]. After Zebeli’s and Ametaj's laboratories introduced this method to dairy cattle and achieved beneficial results in terms of enhanced milk fat production and reduced risk of inflammation [6,7], the advantages of treating cereals with LA have gradually been recognized. Compared with mechanical processing methods such as steam flaking and dry rolling, LA treatment offers economic, safe, and operational benefits. Deckardt et al [8] observed fewer rumen protozoa and enhanced *in vitro* fiber digestion in barley treated with 1% LA than in untreated barley. In a later experiment by Yang et al [4], beef cattle fed 1% LA-treated corn showed improved health performance and reduced rumen lipopolysaccharide (an inflammatory inducer) levels compared to those of cattle fed untreated corn. It was assumed that the advantage of LA-treated cereal on ruminants can be attributed to the debranching effect of LA, which enables amylopectin (branched-chain) to mimic the structure of amylose (straight-chain) [9] because amylose has a slower digestion rate in the rumen that can lower the fermentation rate and increase the rumen pH [10]. Additionally, LA treatment can increase the ratio of resistant starch (RS), allowing this portion of starch to escape rumen digestion, and can be utilized in the intestine with the assistance of pancreatin [11]. In previous studies, a reduction in the protein content of LA-treated cereals was observed [5,9]. However, the exact mechanism underlying the rumen hydrolysis of LA-treated cereal is unclear. The insufficient knowledge regarding LA-treated cereal nutrient variation and subsequent rumen utilization has piqued our interest. We hypothesized that LA treatment could affect cereal crude protein (CP) and RS and will also affect barley ultrastructure. Such variations will lead to significant changes in *in vitro* fermentation characteristics and metabolome. Thus, the objective of this study was to investigate how LA treatment of cereal affects nutrient components and rumen fermentation dynamics using barley as the test cereal.

## MATERIALS AND METHODS

This study was conducted with the consent of the Committee for Animal Research and Welfare of Gifu University (approval ID: #2021-175). The experimental procedures followed the Guidelines for Proper Conduct of Animal Experiments (Science Council of Japan, 2006) and the Guidelines for Animal Research and Welfare of Gifu University (2008).

### Lactic acid treatment of barley

The barley used in the present study was purchased from Toyohashi Feed Mills Co., Ltd. (Aichi, Japan). Then, the barley was milled and screened through two consecutive stainless-steel sieves with mesh sizes of 2.36 and 1.18 mm, respectively. The barley particles retained on the 1.18 mm screen were utilized in subsequent experiments. For the control treatment, barley was soaked in tap water for 48 h (BA). For the LA treatment, barley was soaked in 5% DL-lactic acid (v/v, dissolved in tap water) for 48 h (BALA) as described previously by Deckardt et al [8] and Martínez et al [12]. To prevent any remaining LA on the barley from influencing the results, the soaked barley was gently rinsed with tap water until the pH reached 7.0 and immediately utilized for the *in vitro* experiment.

### Ruminal inoculum and *in vitro* incubation

As the rumen fluid donors, four healthy Japanese Shiba goats (37.1±0.54 kg) raised on the Yanagido farm of Gifu University were selected, maintained for two weeks before donation providing with 760 g of a forage diet (on an as-fed basis: dry matter [DM consisting of 7% alfalfa hay and 93% oat hay, formulated according to the NRC [13]) per day with free access to water and mineral blocks. On the day of donation, rumen fluid was collected three hours after the morning feeding using an esophageal tube [14]. The collected ruminal fluid was immediately strained through four layers of cheesecloth and mixed with preheated (39°C) artificial saliva at a ratio of 1:2 (ruminal fluid:artificial saliva) under continuous CO2 flushing [12]. For different treatments (BA and BALA) and time intervals (3 h, 6 h, 12 h, 18 h, and 24 h), 40 mL of the mixture and 0.5 g of barley particles were separately injected into four fermentation glass vials, flushed with CO_2_, sealed using a butyl rubber plug and tightened with an aluminum cap. Finally, the samples were fixed on a rotary shaker in a water bath (140 rpm) and incubated at 39°C [12]. At each time interval, vials were immediately removed from the water bath, and fermentation was terminated by standing in ice water for five minutes. The pH of the fermentation fluid (inoculant) was measured using a portable pH meter (MP-220; Mettler-Toledo AG, Greifensee, Switzerland), collected into 2-mL tubes, and stored at −80°C for later analysis. The remaining barley in the vials was collected and rinsed in phosphate buffer saline (pH 7.2) three times and immediately subjected to morphological pretreatment.

### Chemical analysis

Milled barley was oven-dried and ground through a 0.5-mm screen for analysis of DM (934.01), CP (984.13), ash (942.05), ether extract (EE; 920.39) and acid detergent lignin (ADL; 973.18) according to the AOAC methods [15]. The acid detergent fiber expressed exclusive of residual ash (ADFom) and neutral detergent fiber with heat-stable amylase expressed exclusive of residual ash (aNDFom) were analyzed according to Van Soest et al [16]. The cellulose content was calculated as ADFom–ADL. The non-fiber carbohydrate content was calculated as 100–aNDFom–CP–EE–ash. Water-soluble carbohydrate levels were measured by calculating the reducing sugar content after the anthrone reaction according to Koehler [17]. For starch determination, barley was freeze-dried and ground to 0.5 mm. Then, the total starch content, amylose content, and RS content were measured using the commercially available K-TSTA, K-AMYL, and K-RSTAR kits (Megazyme, Wicklow, Ireland), respectively. For short-chain fatty acid (SCFA) analysis, the inoculant was extracted into 1.5-mL sampling tubes and analyzed using UPLC-MS (Xevo QToF; Waters, Milford, MA, USA) according to our previous study [18]. The methane yield was calculated considering 90% hydrogen recovery according to Moss et al [19] using the following formula: CH_4_ (mmol/L) = 0.45×acetate (mmol/L) −0.275×propionate (mmol/L) + 0.4×butyrate (mmol/L). Ammonia nitrogen was determined with the phenol-hypochlorite method using an automatic microplate reader. All the nutrients were analyzed in quadruplicate.

### Metabolomic analysis

For metabolome analysis, 500 μL of inoculant was diluted in 990 μL of methanol, and 10 μL of myricetin (1 mg/mL diluted in methanol) was added as the internal standard. Then, the inoculants were vortexed for 20 min, chilled at −20°C for 30 min, and centrifuged at 16,000 rpm at 4°C for 15 min. Finally, the supernatant was recovered for LC-MS analysis. The untargeted metabolome was analyzed using the same UPLC-MS instrument equipped with a C18 analytical column (ACQUITY UPLC BEH C18; 2.1 mm×100 mm; Waters, USA). The mobile phases were 0.1% formic acid (A, diluted in UPLC grade ddH_2_O) and acetonitrile (B), with a running gradient of 0 min, 95% A; 3.0 min, 80% A; 9.0 to 13.0 min, 5% A; and 13.0 to 13.10 min, 95% A and held at 95% until reaching 16 min. The flow rate was 0.4 mL/min, and the injection volume was 5.0 μL. Electrospray ionization was performed in positive and negative modes separately. The mass spectrometer used in MS/MS mode was set as follows: capillary voltage, 2.5 kV; in-source collision-induced dissociation voltage, 30 V; desolvation temperature, 500°C; and source temperature, 150°C. The MS_1_ and MS_2_ data were acquired in MSE mode at 50 to 1,000 m/z for 0.5 s per scan. The low and high collision energies were set at 6 V and ramped from 15 to 45 V for both positive and negative ionization.

The raw LC-MS data were converted into the .abf format using the AnalysisBaseFile Conventor (Reifycs, Inc., Tokyo, Japan) and processed using MSDIAL (Ver. 4.9). The detected ions were matched with the in-house database MSDIAL (Ver. Aug. 21st, 2022). Metabolites with a total score above 700 were considered metabolites. The results from MSDIAL were subsequently transformed into the .csv format, calculated with MetaboAnalyst (ver. 5.0; https://www.metaboanalyst.ca/), normalized and log-transformed [20]. Normalized data were analyzed with the SIMCA software package (Ver. 18; MKS Data Analytics Solutions, Umeå, Sweden) for supervised orthogonal partial least-squares discriminant analysis (OPLS-DA) and permutation tests of the OPLS-DA results. The first principal component of the variable importance in the projection (VIP) value, Student’s *t*-test, and log_2_-fold change (log_2_FC) were calculated. Metabolites with VIP values >1.0, p-values <0.05, and log_2_FC >1.0 or <–1.0 were recognized as differentially accumulated metabolites between the BA and BALA groups. Finally, the differentially accumulated metabolites were analyzed using ‘Pathway Analysis’ embedded in MetaboAnalyst, and the validation of the affected pathways was based on the Kyoto encyclopedia of genes and genomes (KEGG) database.

### Morphological analysis

The dynamic variation in barley morphology during fermentation was visualized using scanning electron microscopy (SEM; S-4800; Hitachi, Tokyo, Japan). The pretreatment process for SEM was as follows: i) The barley particles were fixed in the 1st phosphate buffer (0.2 M; containing 0.5% glutaraldehyde and 0.15% ruthenium red, pH 7.2) for two hours. ii) Then, the particles were transferred to the 2nd phosphate buffer (0.2 M; containing 5% glutaraldehyde and 0.05% ruthenium red, pH 7.2) for two hours. iii) Subsequently, the buffer was removed, and the particles were washed three times with 20 min intervals with the 3rd phosphate buffer (0.2 M; containing 0.05% ruthenium red, pH 7.2). iv) Furthermore, the particles were dehydrated using increasing concentrations of ethanol (10%, 20%, 30%, 50%, 70%, 90%, and 99.5%) at 15 min intervals [12]. Finally, the particles were freeze-dried, placed on aluminum stubs, sputter-coated with osmium, and analyzed using SEM.

### Statistical analysis

The data from the present study were analyzed with the linear mixed model using SPSS software (v20.0; IBM, Armonk, NY, USA) according to the following model: Y_il_ = μ+G_i_+R_j_ +e_ij_, where Y_ij_ represents the dependent variable, μ represents the overall mean, G_i_ represents the fixed effect of cereals, R_j_ represents the random effect of ruminal fluid held within a bottle, and e_ij_ represents the random residual effect. For different time intervals, the data were analyzed as repeated measurements. The fermentation characteristics are presented as the mean±standard error of the mean. The nutrients in LA-treated and untreated barley were measured during laboratory work, and the results are presented as the means± standard deviations. Differences in the means were compared using *t*-tests, and a p-value lower than 0.05 was considered statistically significant. All indices were measured in quadruplicate.

## RESULTS

### Nutrient components of barley

The DM, CP, ash, and water-soluble carbohydrate contents of BALA were lower than those of BA (p<0.05; Table 1). In contrast, the RS and non-fiber carbohydrate contents in the BALA group were higher than those in the BA group (p<0.05). LA treatment of barley resulted in little difference in the contents of EE, NDFom, ADFom, cellulose, total starch, and amylose (p>0.05).

### Variations in fermentation characteristics

**Figure 1:**
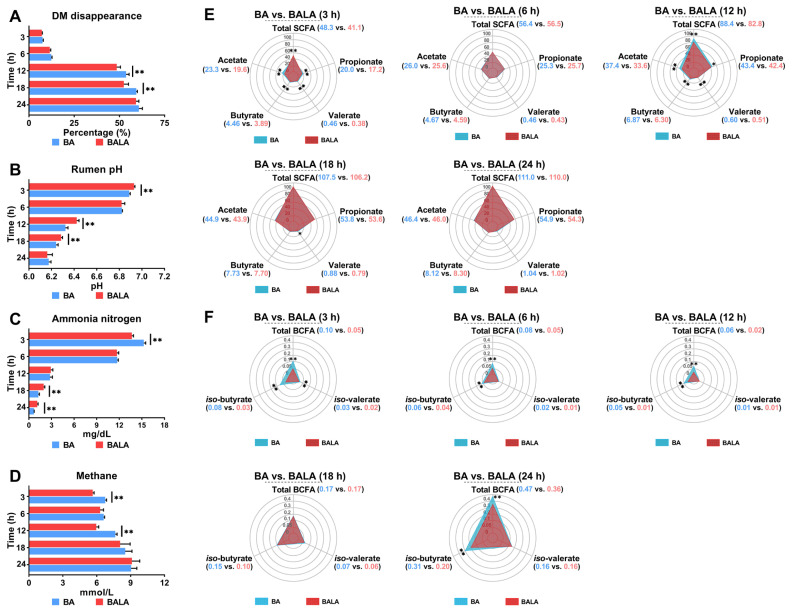
Fermentation characteristics, including dry matter (DM) disappearance (A), rumen pH (B), ammonia nitrogen (C), methane emission (D), short-chain fatty acids (E), and branched-chain fatty acids (F), at different time intervals. The bars, triangles and pentagons marked in blue represent the fermentation of barley steeped with tap water for 48 h (BA), and those marked in red represent the fermentation of barley steeped with 5% lactic acid (dissolved in tap water) for 48 h (BALA). Significance was defined using asterisks (one asterisk indicates p<0.05, two asterisks denote p<0.01).

In the present study, DM disappearance was greater in the BA group than in the BALA group at 12 h and 18 h (p<0.01; Figure 1A), with few differences observed at 3 h, 6 h, or 24 h (p>0.05). The inoculant pH of the BALA group was greater than that of the BA group at 3 h, 12 h, and 18 h (p<0.01; Figure 1B) but exhibited negligible differences at 6 h and 24 h (p>0.05). The NH_3_-N concentration in the BALA was lower at 3 h but greater at 18 and 24 h than that in the BA (p<0.01; Figure 1C); however, the difference was not evident at 6 h and 12 h (p>0.05). Methane emissions from BALA were lower than those from BA at 3 h and 12 h (p<0.01; Figure 1D) but exhibited little difference at 6 h, 18 h, and 24 h (p> 0.05). The concentrations of total SCFAs, acetate, propionate, and butyrate were lower in the BALA group than in the BA group at 3 and 12 h (p<0.05; Figure 1E). However, no difference in the concentrations of these fatty acids was observed between the BA and BALA groups at 6 h, 18 h, and 24 h (p> 0.05). The variation in the valerate concentration followed a similar trend to that of the other SCFAs but was lower at 18 h in the BALA group than in the BA group (p<0.05). Consistent decreases in total BCFA and isobutyrate levels were noted in the BALA group compared with those in the BA group at 3 h, 6 h, 12 h, and 24 h (p<0.01; Figure 1E). The concentration of isovalerate in the BALA group was lower than that in the BA group only at 3 h (p<0.01) but was similar between the BA and BALA groups at other times (p>0.05).

### Multivariate and pathway variations

**Figure 2:**
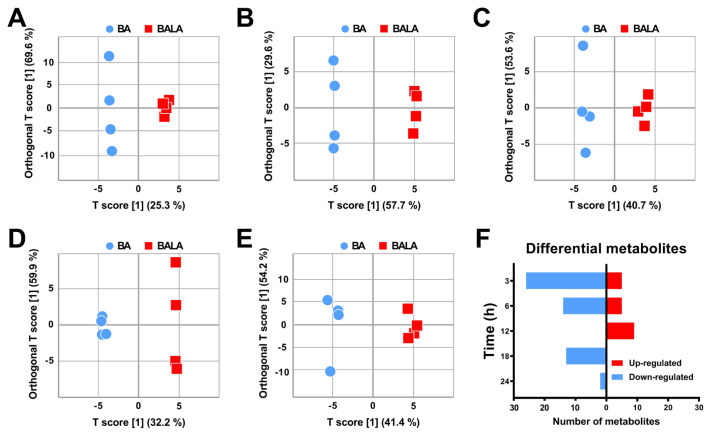
Orthogonal partial least squares discriminant analysis (OPLS-DA) of the untreated barley (BA) group vs. the 5% lactic acid-treated barley (BALA) group at 3 (A), 6 (B), 12 (C), 18 (D), and 24 (E) h of fermentation and the metabolic profile (F) at different time intervals.

A total of 544 MS peaks were identified from positive and negative ionization of the inoculant samples. These peaks corresponded to 58 metabolites (Supplementary Table S1). The orthogonal partial least squares discriminant analysis (OPLS-DA) score plots of all samples at different fermentation time intervals were located in the 95% Hotelling T-squared ellipse (Figure 2A–2E). As shown in the figure, there were apparent plot separations between the BA and BALA groups at all fermentation times. As visualized in the histogram (Figure 2F), four metabolites were upregulated and 21 metabolites were downregulated in the BALA group compared with the BA group at 3 h. In addition, two metabolites were upregulated and 13 metabolites were downregulated in the BALA group compared with the BA group at 6 h. Moreover, eight metabolites were upregulated at 12 h, 12 metabolites were downregulated at 18 h, and only two metabolites were downregulated at 24 h.

**Figure 3:**
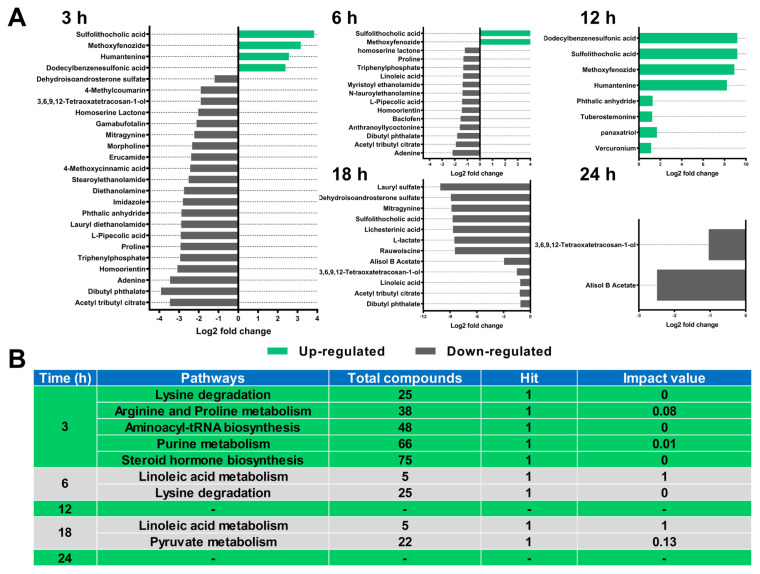
Differential metabolites (A) and affected metabolic pathways (B) of 5% lactic acid-treated barley (BALA) vs. the untreated barley (BA) group. Pathway data were derived by integrating differential metabolites into the bovine metabolome database (BMDB) and Kyoto encyclopedia of genes and genomes (KEGG) database. There were too few differential metabolites identified at 12 and 24 h of fermentation to cluster into specific KEGG metabolic pathways, and these were denoted as ‘-’.

After validation, the major differential metabolites between BA and BALA at different fermentation time intervals are shown in Figure 3A. Subsequently, mapping of the differential metabolites using the KEGG database showed that several pathways were significantly affected (Figure 3B). For instance, at 3 h, compared with BA, BALA had lower concentrations of adenine (log_2_FC = −3.46); proline (log_2_FC = −2.93); L-pipecolic acid (log_2_FC = −2.92), a catabolite of lysine; and dehydroisoandrosterone sulfate (log_2_FC = −1.20), which is a steroid produced by the liver. These differential metabolites were clustered into pathways involved in lysine degradation, arginine and proline metabolism, aminoacyl-tRNA biosynthesis, purine metabolism, and steroid hormone biosynthesis. At 6 h, BALA had lower concentrations of L-pipecolic acid (log_2_FC = −1.42), linoleic acid (log_2_FC = −1.34), and proline (log_2_FC = −1.31), and these metabolites were correlated with linoleic acid metabolism and lysine degradation pathways. At 18 h, BALA had lower concentrations of linoleic acid (log_2_FC = −1.14) and L-lactate (log_2_FC = −8.52), and these two metabolites are involved in linoleic acid metabolism and pyruvate metabolism, respectively. Compared with fermentation at 3 h, 6 h, and 18 h, fermentation at 12 h and 24 h resulted in fewer differential metabolites, such as sulfolithocholic acid (12 h; log_2_FC = 9.18), a type of bile acid; dodecylbenzene sulfonic acid (12 h; log_2_FC = 9.19), a cell signaling molecule; and 3,6,9,12-tetraoxatetracosan-1-ol (24 h; log_2_FC = −1.04), an organic compound that exists in the blood. Unfortunately, these metabolites did not cluster into any metabolic pathway.

### Morphological structure of barley cereals during fermentation

**Figure 4:**
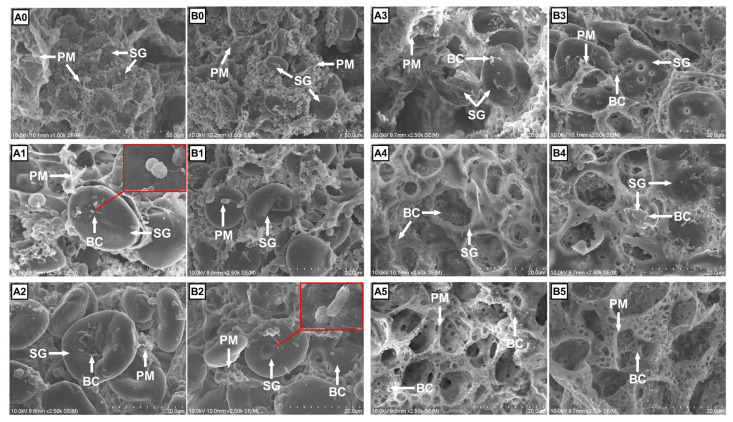
Morphological features (2,500×) of untreated barley (BA; A) and 5% lactic acid-treated barley (BALA; B) viewed by scanning electron microscopy at 0 (A0, B0), 3 (A1, B1), 6 (A2, B2), 12 (A3, B3), 18 (A4, B4), and 24 (A5, B5) h of fermentation. Bacterial cells began to appear on the scanning electron microscopy (SEM) image at 3 h for BA but at 6 h for BALA. Larger images are shown in Supplementary Figure S1. BC, bacterial cells; SG, starch granule; PM, proteinaceous matrix.

The white, protein-like cover on the ultrastructures of barley and LA-treated barley before (0 h) and during fermentation (3 to 24 h) observed by SEM was defined as the ‘proteinaceous matrix (PM)’, a term used by Martínez et al [12]. The difference in the ultrastructure of barley and LA-treated barley was notable since the onset of fermentation (Figure 4). At 0 h (Figure 4 A0, B0), the most evident variation induced by LA treatment was the reduction in the PM that surrounded the starch granule (SG) in the BALA compared with that in the BA. However, minimal noticeable differences in the morphological structure of the SG were noted between the BALA and BA. After 3 h (Figure 4 A1, B1), both BA and BALA had an exposed SG due to the degradation of PM. However, clustered bacterial cells began colonizing the SG of BA but not BALA. At 6 h (Figure 4 A2, B2), the hydrolysis of the PM was very rapid compared with that of the SG on both BA and BALA, and less PM was observed on the BA than on the BALA. Moreover, clustered bacterial cells were also apparent on the SG of the BALA group. After 12 h (Figure 4 A3, B3), the number of bacteria on the SG surface decreased in the BA and BALA groups, and cavities began to appear on the SGs. BA was more digested than BALA because of the larger diameter of the cavities. After 18 h (Figure 4 A4, B4), the SGs of BA and BALA nearly all degraded. However, SG can still be observed on the BALA. Finally, at 24 h (Figure 4 A5, B5), no SG could be observed on the BA or BALA; only net-like structures and bacterial cells remained. There was no distinction between the BA and BALA.

## DISCUSSION

### Impact of lactic acid on barley nutrient components

Several studies have indicated that LA-treated cereals affect rumen fermentation by modifying nutrient properties [7,21]. Indeed, we observed alterations in both the nutrient compositions and ultrastructures of barley after LA treatment. The lower protein content in the BALA group than in the BA group aligns with the report of Dailey et al [5], who indicated protein leakage into the steeping solution during 0.5% LA treatment. Additionally, the SEM image showed a decrease in the PM content on the BALA compared with that on the BA. The mechanism of protein decrease was probably the softening of the PM during steeping [22] and the solubilization of cereal proteins in LA [23]. A previous study suggested the activation of endogenous protease during LA treatment of corn [5]. However, in that study, protein solubilization occurred in untreated corn, and similar protein contents were detected in the corn kernels treated with either L- or DL-lactic acid. Of note, although LA possesses antimicrobial capacity [24], complete depletion of microbes is impossible. Therefore, microbial degradation of PM could also be considered. To draw a precise conclusion, more data are still needed. The decrease in the water-soluble carbohydrate content of the BALA group compared to that of the BA group was believed to be induced by LA activation of endogenous enzymes such as β-glucanase [25]. The lower ash content can be attributed to the decreasing effect of LA on barley mineral content [8], as a previous study revealed a decreased phosphorus level after treating corn with 25 g/kg LA for 48 h [9].

The greater RS content in the BALA group compared with the BA group aligns with our hypothesis that LA treatment may influence specific cereal nutrients. This finding is also consistent with previous studies, which aimed to increase the flow of RS to the hindgut through LA treatment to enhance host energy utilization [26]. The increase in the RS content was induced by a series of irregular reactions, such as cross-linking and depolymerization (as reviewed by Sajilata et al [27]), which ultimately decreased the susceptibility of this part of the starch to rumen degradation. Our observation is consistent with the results of Vötterl et al [9], who reported that the aNDFom content remained stable after LA treatment, but contrasts with the findings of Harder et al [28], who reported a lower NDF content after treating barley with 5% LA for 24 h. The discrepancies may be attributed to variations in LA concentrations. Nevertheless, the limited data do not permit us to confirm the precise mechanism underlying these alterations.

### Variations in fermentation characteristics

Following the alterations in nutrient contents, the *in vitro* fermentation of barley was affected to a certain extent. Although LA treatment reduced the protein content of BALA, the simultaneous decrease in DM content may attenuate the discrepancies in DM loss between BA and BALA during the intensive protein fermentation period from 0 h to 6 h [3]. Therefore, it seems reasonable that the two groups had similar DM disappearances at 3 h and 6 h. On the other hand, the lower DM loss in BALA compared with BA at 12 h and 18 h can be attributed to the higher RS content in BALA, as this type of starch generally degrades more slowly than other starch types in the rumen [29]. More intuitively, differences in DM loss at 12 h and 18 h can be supported by the remaining starch granules evident in the comparison of SEM images between the BALA group and BA group. The comparable DM disappearance between the BA and BALA groups at 24 h may be attributed to the depletion of proteins and starch, not the remaining cell wall components, such as cellulose [12].

The pH increase observed at 3 h, 12 h, and 18 h in the BALA group was consistent with the observation of Iqbal et al [7], where dairy cows fed 1% LA-treated barley exhibited elevated rumen pH. However, the beneficial effect on pH is likely multifactorial. This was because the rumen degradation rate of protein is often faster than that of starch, and protein is generally exhausted at approximately 12 h of fermentation [3]. Therefore, the higher pH at 3 h was attributed to the lower protein content in BALA in response to BA, whereas the elevated pH at 12 and 18 h was likely induced by the higher RS content in BALA than in BA. Similar to the DM disappearance, the absence of a pH difference after 24 h may be attributed to the depletion of starch and the onset of fiber fermentation [12]. The similar aNDFom and ADFom values thus contributed to the stable pH between the BA and BALA groups to a large extent.

The lower NH_3_-N concentration in the BALA group than in the BA group at 3 h accurately reflected the lower protein content induced by LA treatment. However, the mechanism underlying the similar pH levels and NH_3_-N concentrations at 6 h between the BA and BALA groups is unclear. One possible reason is that a considerable amount of PM was observed in the SEM images of both the BA and BALA samples, and another is the potential of LA to decrease soluble protein levels, mainly those of albumin and globulin, in cereals [30,31]. This could be attributed to LA selectively decreasing the levels of fast-degrading proteins, such as albumin and globulin. In contrast, other proteins, such as hordein and glutelin [32], remained unaffected.

Iqbal et al [6] reported no effect of 0.5% LA on total rumen SCFAs during diurnal variation but did observe lower total SCFA levels at 2 h and 4 h postfeeding. In partial agreement with this report, we noticed that LA treatment promoted a decrease in total SCFAs in BA and BALA samples not only at 3 h but also at 12 h of fermentation. Rumen SCFAs are produced mainly from the microbial fermentation of carbohydrates rather than proteins [33]. Therefore, the lower total SCFA levels in the BALA group at 3 h of fermentation in our study can be attributed only to the LA-induced decrease in the water-soluble carbohydrate content. Similarly, the lower total SCFA content at 12 h in the BALA group was caused by the greater RS content. In contrast with the studies that only observed lower acetate but were in line with those of Harder et al [34], the decrease in the contents of all individual SCFAs may be due to the higher LA concentration (5% LA in the present study and 0.5 and 1% in Iqbal et al [6] and Yang et al [4], respectively), which accelerated the decrease in the water-soluble carbohydrate content or increase in the RS content. One compelling result was that LA-treated barley significantly reduced methane emission during the most intensive fermentation periods of protein (approximately 3 h of fermentation) and starch (12 h to 18 h of fermentation), as postulated by Humer and Zebeli [26]. The variation in methane concentration can be directly attributed to the decrease in total and individual SCFAs induced by LA treatment possibly because either LA treatment reduces the substrate available for methanogens to generate methane or reduces the amount of acetate synthesized, leading to a reduction in the amount of metabolic hydrogen needed for methane production [35]. However, as the methane concentration in our study was measured indirectly by calculation, validation of this conclusion still requires additional *in vivo* data.

The lower total BCFA concentration in the BALA group, especially at 3 h, corresponded with a reduced NH_3_-N concentration. This reduction can be induced either by LA reducing protein content [9] or by LA protecting amino acids from degradation [36]. The lack of a difference in *iso*-valerate content between the BA and BALA groups but a difference in *iso*-butyrate content was in accordance with the results of Mickdam et al [21] because LA-treated cereals affect the bacterium *Ruminococcus albus*, which is more correlated with iso-butyrate than iso-valerate in the rumen [37]. Unfortunately, limited information does not allow us to explain the sudden increase in isobutyrate content in the BA group after 24 h of fermentation. We speculate that this result was related to microbial differences between the BA and BALA groups because *iso*-fatty acids are essential components for some bacteria during fiber degradation.

### Dynamics of the fermentation metabolome

LA treatment decreased the barley CP content, reducing the volume of NH_3_-N produced during *in vitro* fermentation. In response, adenine, L-pipecolic acid, and proline were lower in the BALA group than in the BA group at 3 h. As adenine is an intermediate of purine metabolism [38], its decreased content in the rumen implies suppressed synthesis of microbial proteins [39]. Moreover, the reduction in adenine content corresponded well with the decrease in CP and NH_3_-N levels in BALA, as microbial protein synthesis primarily relies on dietary nitrogen sources such as CP. On the other hand, L-pipecolic acid is the product of lysine degradation [40]. The simultaneous decrease in L-pipecolic acid and proline levels indicated a decrease in upstream protein utilization. Therefore, it can be concluded that LA treatment of barley predominantly affects rumen fermentation at 3 h by changing protein utilization.

As fermentation progressed and barley proteins were rapidly exhausted (indicated by lower PM content on SEM images at 6 h than at 3 h), fewer differential metabolites and affected pathways were observed at 6 h compared to 3 h. Only the lysine degradation pathway remained affected in terms of protein utilization. The reduced linoleic acid content in the BALA group compared to that in the BA group indicated attenuated linoleic acid metabolism. However, few reports have evaluated the relationship between LA treatment of cereals and linoleic acid in ruminants. Notably, Wood et al [41] indicated that linoleic acid can efficiently provide energy to the host, and experiments conducted on pigs revealed greater plasma linoleic acid levels in the energy-deficient group than in the energy-sufficient group [42]. Combined with the report that enhanced energy status was observed in dairy cattle consuming LA-treated barley [29], we suggest that the reduced linoleic acid level released in the rumen is beneficial, but the mechanism involved remains to be determined. At 12 h, no pathways could be attributed to protein utilization or starch fermentation. Furthermore, the difference in pyruvate metabolism at 18 h in the BALA group agreed with the results of Yang et al [4], who observed lower rumen pyruvate contents in steers fed 1% LA-treated corn than in steers fed untreated corn. As pyruvate is the intermediate product of starch digestion [4], the attenuation of pyruvate metabolism clearly showed that starch in LA-treated barley escaped from rumen digestion. Finally, at 24 h, no pathways were affected, and only a few differential metabolites were observed. This, coupled with similar DM disappearance, inoculant pH, and ultrastructures between BA and BALA, suggested the complete fermentation of protein and starch. This result was unexpected because the purpose of using LA-treated cereal was to save more nutrients from the rumen for utilization in the intestine. Thus, to guarantee better utilization of cereals in the intestine, factors that slow the rumen passage rate of LA-treated cereal, such as adding another viscous feedstuff or decreasing DM intake, should be avoided.

**Figure 5:**
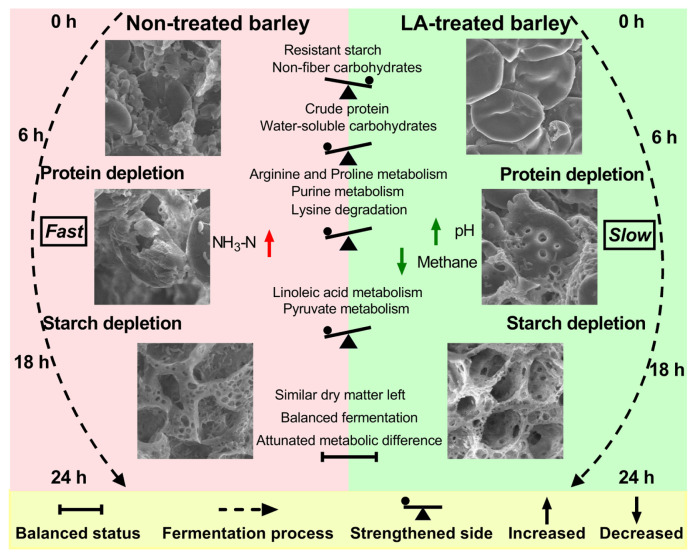
Fate of LA-treated barley (BALA) and untreated barley (BA) degradation in the rumen. Lactic acid (LA) treatment first alters the nutrient composition of barley, primarily by increasing the content of resistant starch and nonfiber carbohydrates and decreasing the content of crude protein and water-soluble carbohydrates. BALA and BA degradation in the rumen causes marked discrepancies in fermentation characteristics, the rumen metabolome, and the barley ultrastructure. However, these discrepancies diminished after degradation for 24 h. In general, BA degraded faster than BALA.

A summary of the effects of LA treatment on barley nutrients and morphological structure, as well as differences in fermentation characteristics and metabolomes, between BA and BALA is shown in Figure 5. Briefly, LA treatment modified barley by increasing the RS and non-fiber carbohydrate contents and decreasing the CP and water-soluble carbohydrate contents. These alterations subsequently led to significant differences in fermentation characteristics (such as pH, NH_3_-N, and methane), metabolome (by attenuating specific metabolic pathways), and ultrastructure.

## CONCLUSION

The consistency of our results with previous *in vitro* and *in vivo* data underscores the strong representativeness of our data for practical ruminant responses. On the other hand, the variation in fermentation characteristics, such as inoculant pH and SCFAs, combined with the altered metabolic pathways observed in the BALA group compared to those in the BA group, supports our hypothesis that LA treatment of cereals can alter rumen fermentation and metabolism by modifying cereal nutrient contents such as CP and RS. More importantly, we provided the first view of metabolomic and morphological changes in both LA-treated and untreated barley during a 24-h fermentation period, offering a valuable reference for future in-depth investigations.

## Figures and Tables

**Table 1:** Nutrient composition of cereals (% of dry matter bases; presented as the mean with standard deviation from quadruplicate measurements)

Items	BA	BALA	p-value
Dry matter	82.36±0.05	79.88±0.12	<0.001
Crude protein	10.13±0.06	9.11±0.08	<0.001
Ether extract	1.88±0.05	1.80±0.11	0.509
aNDFom	11.30±0.22	11.67±0.40	0.699
ADFom	3.55±0.25	3.67±0.23	0.588
ADL	0.30±0.19	0.32±0.18	0.899
Cellulose	3.34±0.03	3.35±0.06	0.845
Ash	2.17±0.16	1.54±0.07	<0.001
Total starch	60.97±1.89	62.43±1.60	0.345
Amylose	24.75±1.61	23.95±1.43	0.543
Resistant starch	0.79±0.11	3.84±0.18	<0.001
Non-fiber carbohydrate	74.48±0.24	76.31±0.49	0.001
Water-soluble carbohydrate	0.51±0.05	0.40±0.02	0.010

BA, barley grain; BALA, 5% lactic acid-treated barley grain; aNDFom, neutral detergent fiber with heat-stable amylase expressed exclusive of residual ash; ADFom, acid detergent fiber expressed exclusive of residual ash; ADL, acid detergent lignin.

## SUPPLEMENTARY MATERIAL

**Supplementary Table S1**. Parameters of metabolites from positive and negative ionization at different fermentation time intervals

**Supplementary Figure S1**. Morphological features (2,500×) of barley (A) and 5% lactic acid-treated barley (B) viewed by scanning electron microscopy at 0 (A0, B0), 3 (A1, B1), 6 (A2, B2), 12 (A3, B3), 18 (A4, B4), and 24 (A5, B5) h of fermentation.

## References

[1] Khafipour E, Krause DO, Plaizier JC (2009). A grain-based subacute ruminal acidosis challenge causes translocation of lipopolysaccharide and triggers inflammation. J Dairy Sci.

[2] Zebeli Q, Mansmann D, Steingass H, Ametaj BN (2010). Balancing diets for physically effective fibre and ruminally degradable starch: A key to lower the risk of sub-acute rumen acidosis and improve productivity of dairy cattle. Livest Sci.

[3] Nikkhah A (2012). Barley grain for ruminants: a global treasure or tragedy. J Anim Sci Biotechnol.

[4] Yang Y, Dong G, Wang Z, Liu J, Chen J, Zhang Z (2018). Treatment of corn with lactic acid or hydrochloric acid modulates the rumen and plasma metabolic profiles as well as inflammatory responses in beef steers. BMC Vet Res.

[5] Dailey OD, Dowd MK, Mayorga JC (2000). Influence of lactic acid on the solubilization of protein during corn steeping. J Agric Food Chem.

[6] Iqbal S, Zebeli Q, Mazzolari A (2009). Feeding barley grain steeped in lactic acid modulates rumen fermentation patterns and increases milk fat content in dairy cows. J Dairy Sci.

[7] Iqbal S, Terrill SJ, Zebeli Q (2012). Treating barley grain with lactic acid and heat prevented sub-acute ruminal acidosis and increased milk fat content in dairy cows. Anim Feed Sci Technol.

[8] Deckardt K, Metzler-Zebeli BU, Zebeli Q (2016). Processing barley grain with lactic acid and tannic acid ameliorates rumen microbial fermentation and degradation of dietary fibre in vitro. J Sci Food Agric.

[9] Vötterl JC, Zebeli Q, Hennig-Pauka I, Metzler-Zebeli BU (2019). Soaking in lactic acid lowers the phytate-phosphorus content and increases the resistant starch in wheat and corn grains. Anim Feed Sci Technol.

[10] Beauchemin KA, McGinn SM (2005). Methane emissions from feedlot cattle fed barley or corn diets1. J Anim Sci.

[11] McCleary BV, Monaghan DA (2002). Measurement of resistant starch. J AOAC Int.

[12] Martínez TF, McAllister TA, Wang Y, Reuter T (2006). Effects of tannic acid and quebracho tannins on in vitro ruminal fermentation of wheat and corn grain. J Sci Food Agric.

[13] National Research Council (NRC) (2007). Nutrient requirements of small ruminants: sheep, goats, cervids, and new world camelids.

[14] Tian K, Liu J, Sun Y (2019). Effects of dietary supplementation of inulin on rumen fermentation and bacterial microbiota, inflammatory response and growth performance in finishing beef steers fed high or low-concentrate diet. Anim Feed Sci Technol.

[15] Horwitz W (2005). AOAC International Official methods of analysis of AOAC International.

[16] Van Soest PJ, Robertson JB, Lewis BA (1991). Methods for dietary fiber, neutral detergent fiber, and nonstarch polysaccharides in relation to animal nutrition. J Dairy Sci.

[17] Koehler LH (1952). Differentiation of carbohydrates by anthrone reaction rate and color intensity. Anal Chem.

[18] Aldian D, Harisa LD, Mitsuishi H, Tian K, Iwasawa A, Yayota M (2023). Diverse forage improves lipid metabolism and antioxidant capacity in goats, as revealed by metabolomics. Animal.

[19] Moss AR, Jouany JP, Newbold J (2000). Methane production by ruminants: its contribution to global warming. Ann Zootech.

[20] Pang Z, Zhou G, Ewald J (2022). Using MetaboAnalyst 5.0 for LC–HRMS spectra processing, multi-omics integration and covariate adjustment of global metabolomics data. Nat Protoc.

[21] Mickdam E, Khiaosa-ard R, Metzler-Zebeli BU (2017). Modulation of ruminal fermentation profile and microbial abundance in cows fed diets treated with lactic acid, without or with inorganic phosphorus supplementation. Anim Feed Sci Technol.

[22] Zhu JH, Haase NU, Kempf W (1990). Investigations on the laboratory scale separation of mung bean starch. Starch-Stärke.

[23] Dailey OD (2002). Effect of lactic acid on protein solubilization and starch yield in corn wet-mill steeping: a study of hybrid effects. Cereal Chem.

[24] Vandenbergh PA (1993). Lactic acid bacteria, their metabolic products and interference with microbial growth. FEMS Microbiol Rev.

[25] Skrede G, Herstad O, Sahlstrøm S, Holck A, Slinde E, Skrede A (2003). Effects of lactic acid fermentation on wheat and barley carbohydrate composition and production performance in the chicken. Anim Feed Sci Technol.

[26] Humer E, Zebeli Q (2017). Grains in ruminant feeding and potentials to enhance their nutritive and health value by chemical processing. Anim Feed Sci Technol.

[27] Sajilata MG, Singhal RS, Kulkarni PR (2006). Resistant starch-a review. Compr Rev Food Sci Food Saf.

[28] Harder H, Khol-Parisini A, Zebeli Q (2015). Modulation of resistant starch and nutrient composition of barley grain using organic acids and thermal cycling treatments. Starch-Stärke.

[29] Iqbal S, Zebeli Q, Mazzolari A, Dunn SM, Ametaj BN (2010). Feeding rolled barley grain steeped in lactic acid modulated energy status and innate immunity in dairy cows. J Dairy Sci.

[30] Serna-Saldívar SO, Mezo-Villanueva M (2003). Effect of a cell-wall-degrading enzyme complex on starch recovery and steeping requirements of sorghum and maize. Cereal Chem.

[31] Tian KE, Luo G, Aldian D, Yayota M (2024). Treatment of corn with lactic acid delayed in vitro ruminal degradation without compromising fermentation: a biological and morphological monitoring study. Front Vet Sci.

[32] Hellebois T, Gaiani C, Planchon S, Renaut J, Soukoulis C (2021). Impact of heat treatment on the acid induced gelation of brewers’ spent grain protein isolate. Food Hydrocoll.

[33] Pereira AM, de Lurdes Nunes Enes Dapkevicius M, Borba AES (2022). Alternative pathways for hydrogen sink originated from the ruminal fermentation of carbohydrates: which microorganisms are involved in lowering methane emission?. Anim Microbiome.

[34] Harder H, Khol-Parisini A, Metzler-Zebeli BU, Klevenhusen F, Zebeli Q (2015). Treatment of grain with organic acids at 2 different dietary phosphorus levels modulates ruminal microbial community structure and fermentation patterns in vitro. J Dairy Sci.

[35] Newbold CJ, de la Fuente G, Belanche A, Ramos-Morales E, McEwan NR (2015). The role of ciliate protozoa in the rumen. Front Microbiol.

[36] Petrova P, Petrov K (2020). Lactic acid fermentation of cereals and pseudocereals: ancient nutritional biotechnologies with modern applications. Nutrients.

[37] Macfarlane S, Macfarlane GT (2003). Regulation of short-chain fatty acid production. Proc Nutr Soc.

[38] Tie S, Zhang L, Li B (2023). Effect of dual targeting procyanidins nanoparticles on metabolomics of lipopolysaccharide-stimulated inflammatory macrophages. Food Sci Hum Wellness.

[39] Chen XB, Gomes MJ (1992). Estimation of microbial protein supply to sheep and cattle based on urinary excretion of purine derivatives: an overview of the technical details.

[40] Broquist HP (1991). Lysine-pipecolic acid metabolic relationships in microbes and mammals. Annu Rev Nutr.

[41] Wood JD, Richardson RI, Nute GR (2004). Effects of fatty acids on meat quality: a review. Meat Sci.

[42] Liu H, Chen Y, Ming D (2018). Integrative analysis of indirect calorimetry and metabolomics profiling reveals alterations in energy metabolism between fed and fasted pigs. J Anim Sci Biotechnol.

