# Supplementary material for: Metabolomic and morphologic surveillance reveals the impact of lactic acid-treated barley on in vitro ruminal fermentation
Source: Anim Biosci. 2024 May 7;37(11):1901–12. doi: 10.5713/ab.23.0550 (PMC11541031; doi:10.5713/ab.23.0550)
Supplement: Supplementary file 1 [file ab-23-0550-Supplementary-Table-1.pdf]

Supplementary Table S1

Parameters of metabolites from positive and negative ionization at different fermentation time intervals.

**Metabolites (BALA vs. BA)**	**VIP**	**log$_2$FC**	***P* value**	**Metabolites (BALA vs. BA)**	**VIP**	**log$_2$FC**	***P* value**
				**3 h**			
Positive ionization				***Negative ionization***			
Dibutyl phthalate	1.18	-3.92	< 0.01	Sulfolithocholic acid	1.81	3.85	< 0.01
Acetyl tributyl citrate	1.19	-3.47	< 0.01	Humantenine	1.78	2.57	< 0.01
Adenine	1.18	-3.46	< 0.01	Methoxyfenozide	1.63	3.17	< 0.01
Homoorientin	1.16	-3.09	< 0.01	Mitragynine	1.80	-2.23	< 0.01
Triphenylphosphate	1.12	-2.95	< 0.01	Dodecylbenzenesulfonic acid	1.34	-1.19	0.03
Proline	1.11	-2.93	< 0.01	Dehydroisoandrosterone sulfate	1.48	2.39	0.04
L-Pipecolic acid	1.16	-2.92	< 0.01	Lichesterinic acid	1.24	1.00	0.07
Lauryl diethanolamide	1.13	-2.90	< 0.01	DL-2-hydroxyvaleric acid	1.14	-0.52	0.10
Phthalic anhydride	1.14	-2.88	< 0.01	LPE 16:0	0.82	0.25	0.27
Imidazole	1.11	-2.81	< 0.01	Lauric acid	0.73	0.19	0.31
Diethanolamine	1.12	-2.75	< 0.01	Myristic acid	0.74	-0.22	0.34
Stearoylethanolamide	1.15	-2.52	< 0.01	Benzenesulfonic acid	0.73	0.25	0.34

4-Methoxycinnamic acid	1.10	-2.44	0.01
Erucamide	1.13	-2.39	0.01
Morpholine	1.05	-2.34	0.02
Gamabufotalin	1.07	-2.11	0.02
Homoserine Lactone	1.00	-2.03	0.04
3,6,9,12-Tetraoxatetracosan-1-ol	1.00	-1.90	0.02
4-Methylcoumarin	1.00	-1.90	0.03
Anthranoyllycoctonine	0.94	-2.22	0.05
Alisol B Acetate	0.86	1.82	0.07
Vercuronium	0.81	-1.58	0.10
Tuberostemonine	0.70	-1.26	0.17
Panaxatriol	0.52	-0.98	0.34
Leupeptin	0.31	-0.35	0.58
Baclofen	0.11	-1.27	0.81

Rauwolscine	0.59	0.16	0.41
Ortophosphate	0.65	0.22	0.44
Thymol-β-D-glucoside	0.51	0.16	0.47
Indoxyl sulfate	0.52	0.14	0.50
Zinniol	0.40	0.12	0.54
Lauryl sulfate	0.44	0.12	0.54
Trifluoroacetic acid	0.32	0.07	0.67
Stearic acid	0.33	0.06	0.68
Undecanedioic acid	0.32	-0.12	0.69
Decanedioic acid	0.24	-0.10	0.75
Azelaic acid	0.22	-0.10	0.76
Ascorbic acid	0.18	-0.14	0.79
2-Hydroxyisocaproic acid	0.12	< 0.01	0.88
Dodecanedioic acid	0.05	-0.07	0.94
Allantoin	0.03	-0.07	0.95

6 h

Positive ionization

Negative ionization

Adenine	1.37	-2.18	< 0.01	Sulfolithocholic acid	2.09	4.73	0.02
Acetyl tributyl citrate	1.30	-1.91	0.01	Methoxyfenozide	1.94	4.32	0.04
Dibutyl phthalate	1.28	-1.81	0.01	Dodecylbenzenesulfonic acid	1.82	3.62	0.07
Anthranoyllycoctonine	1.25	-1.60	0.02	Mitragynine	1.81	-1.39	0.08
Baclofen	1.28	-1.54	0.02	Dehydroisoandrosterone sulfate	1.76	-1.25	0.09
Homoorientin	1.22	-1.44	0.02	Humantenine	1.66	3.52	0.11
L-Pipecolic acid	1.23	-1.42	0.02	Rauwolscine	1.29	-0.58	0.25
N-lauroylethanolamine	1.12	-1.39	0.04	Ascorbic acid	0.92	0.58	0.42
Myristoyl ethanolamide	1.14	-1.37	0.04	L-lactate	0.63	1.78	0.58
Linoleic acid	1.16	-1.34	0.04	Allantoin	0.54	0.85	0.65
Triphenylphosphate	1.24	-1.32	0.04	Myristic acid	0.53	0.68	0.65
Proline	1.11	-1.31	0.04	LPE 16:0	0.49	0.66	0.67
Homoserine lactone	1.10	-1.21	0.04	Ortophosphate	0.48	0.85	0.68
Phthalic anhydride	1.10	-1.36	0.05	DL-2-hydroxyvaleric acid	0.44	0.18	0.70
Lauryl diethanolamide	1.03	-1.11	0.08	Dodecanedioic acid	0.39	0.73	0.74
Imidazole	1.02	-1.16	0.08	Undecanedioic acid	0.37	0.76	0.75
Diethanolamine	1.01	-1.15	0.09	Azelaic acid	0.35	0.82	0.76

Morpholine	0.93	-1.13	0.12	Decanedioic acid	0.34	0.77	0.77
Erucamide	0.93	-0.88	0.13	Indoxyl sulfate	0.32	0.81	0.77
Gamabufotalin	0.91	-1.01	0.13	Stearic acid	0.24	0.87	0.79
Panaxatriol	0.87	0.64	0.16	Lichesterinic acid	0.23	1.49	0.84
4-Methoxycinnamic acid	0.88	-0.89	0.16	Lauryl sulfate	0.23	1.00	0.85
Stearamide	0.91	-0.45	0.17	Trifluoroacetic acid	0.21	1.11	0.86
3,6,9,12-Tetraoxatetracosan-1-ol	0.78	-0.80	0.21	Zinniol	0.15	1.22	0.89
4-Methylcoumarin	0.56	-0.58	0.40	Lauric acid	0.15	1.09	0.90
Alisol B Acetate	0.42	0.32	0.46	Thymol-β-D-glucoside	0.13	1.17	0.92
Tuberostemonine	0.45	0.19	0.49	Benzenesulfonic acid	0.10	1.22	0.93
Leupeptin	0.34	0.06	0.61	2-Hydroxyisocaproic acid	0.06	0.86	0.95
Vercuronium	0.16	-0.05	0.82	Canrenone	0.06	1.38	0.97
				**12 h**			
Positive ionization				***Negative ionization***			
Vercuronium	1.62	1.14	< 0.01	Humantenine	1.29	8.21	0.01
Panaxatriol	1.66	1.68	< 0.01	Methoxyfenozide	1.34	8.91	0.01
Tuberostemonine	1.61	1.24	< 0.01	Sulfolithocholic acid	1.34	9.18	0.01

Phthalic anhydride	1.56	1.28	< 0.01	Dodecylbenzenesulfonic acid	1.31	9.19	0.01
Leupeptin	1.52	0.99	< 0.01	Canrenone	1.06	6.15	0.07
4-Methylcoumarin	1.38	0.69	0.02	Zinniol	1.05	5.99	0.08
Acetyl tributyl citrate	1.30	-0.42	0.04	Lauric acid	1.05	5.98	0.08
Morpholine	1.26	0.44	0.05	Thymol-β-D-glucoside	1.05	5.98	0.08
Alisol B Acetate	1.24	-1.00	0.06	Lauryl sulfate	1.04	5.96	0.08
4-Methoxycinnamic acid	1.15	0.45	0.08	Myristic acid	1.02	6.09	0.09
Lauryl diethanolamide	1.15	0.41	0.09	Benzenesulfonic acid	0.99	5.78	0.10
Diethanolamine	1.13	0.34	0.10	2-Hydroxyisocaproic acid	0.99	5.67	0.10
3,6,9,12-Tetraoxatetracosan-1-ol	1.04	-0.41	0.12	LPE 16:0	0.98	5.98	0.11
Dibutyl phthalate	1.03	-0.29	0.13	Trifluoroacetic acid	0.98	5.76	0.11
Gamabufotalin	0.99	0.28	0.16	Indoxyl sulfate	0.97	5.70	0.11
Adenine	0.66	-0.18	0.36	Azelaic acid	0.96	5.56	0.11
Erucamide	0.81	0.43	0.24	Decanedioic acid	0.96	5.57	0.11
Triphenylphosphate	0.63	0.18	0.38	Undecanedioic acid	0.96	5.59	0.12
L-Pipecolic acid	0.61	0.14	0.42	Stearic acid	0.96	5.62	0.12
Stearamide	0.57	0.44	0.44	Dodecanedioic acid	0.96	5.57	0.12

Imidazole	0.58	0.13	0.45	Ortophosphate	0.96	5.53	0.12
Proline	0.38	0.10	0.60	L-lactate	0.96	5.54	0.12
Baclofen	0.34	0.08	0.66	Allantoin	0.94	5.48	0.13
N-lauroylethanolamine	0.26	0.07	0.72	DL-2-hydroxyvaleric acid	0.93	5.41	0.13
Anthranoyllycoctonine	0.20	0.05	0.75	Lichesterinic acid	0.93	3.81	0.15
Homoorientin	0.19	-0.06	0.78	Ascorbic acid	0.88	5.13	0.15
Myristoyl Ethanolamide	0.17	0.03	0.84	Rauwolscine	0.64	3.98	0.16
Homoserine lactone	0.09	0.02	0.96	Dehydroisoandrosterone sulfate	0.52	3.51	0.44
Linoleic acid	0.03	< -0.01	0.98	Mitragynine	0.49	3.45	0.46
			**18 h**				
Positive ionization				***Negative ionization***			
Alisol B Acetate	1.50	-2.92	< 0.01	Lauryl sulfate	1.25	-10.10	0.01
3,6,9,12-Tetraoxatetracosan-1-ol	1.42	-1.52	< 0.01	L-lactate	1.22	-8.52	0.02
Linoleic acid	1.44	-1.14	< 0.01	Mitragynine	1.21	-8.85	0.02
Acetyl tributyl citrate	1.31	-1.18	< 0.01	Dehydroisoandrosterone sulfate	1.18	-8.91	0.02
Adenine	1.31	-0.99	0.01	Sulfolithocholic acid	1.17	-8.71	0.02
Dibutyl phthalate	1.24	-1.11	0.02	Lichesterinic acid	1.17	-8.68	0.02

Homoorientin	1.21	-0.98	0.02	Rauwolscine	1.16	-8.45	0.03
Homoserine lactone	1.17	-0.96	0.03	DL-2-hydroxyvaleric acid	1.10	-7.10	0.07
Baclofen	1.11	-0.75	0.04	Ascorbic acid	1.00	-7.10	0.08
N-lauroylethanolamine	1.12	-0.77	0.05	Allantoin	0.99	-6.92	0.08
Phthalic anhydride	1.12	-0.76	0.05	Stearic acid	0.98	-6.84	0.08
Myristoyl Ethanolamide	1.11	-0.77	0.05	Ortophosphate	0.98	-6.57	0.08
Proline	1.09	-0.72	0.06	Azelaic acid	0.96	-6.78	0.09
Imidazole	1.05	-0.66	0.07	Thymol-β-D-glucoside	0.96	-6.64	0.09
Anthranoyllycoctonine	1.03	-0.73	0.07	Undecanedioic acid	0.96	-6.75	0.09
Triphenylphosphate	1.03	-0.65	0.08	Benzenesulfonic acid	0.96	-6.56	0.09
Panaxatriol	0.98	0.53	0.10	2-Hydroxyisocaproic acid	0.95	-6.75	0.09
Diethanolamine	0.95	-0.60	0.10	Decanedioic acid	0.96	-6.75	0.09
Morpholine	0.91	-0.56	0.12	Dodecanedioic acid	0.96	-6.86	0.09
L-Pipecolic acid	0.92	-0.58	0.13	Indoxyl sulfate	0.95	-6.62	0.09
Gamabufotalin	0.89	-0.53	0.14	Methoxyfenozide	0.94	-6.59	0.10
4-Methoxycinnamic acid	0.75	-0.42	0.23	Canrenone	0.94	-6.51	0.10
Lauryl diethanolamide	0.74	-0.36	0.23	Trifluoroacetic acid	0.94	-6.60	0.10

Stearamide	0.50	-0.30	0.45	Zinniol	0.93	-6.42	0.10
Erucamide	0.49	-0.30	0.46	Lauric acid	0.93	-6.40	0.11
4-Methylcoumarin	0.40	-0.24	0.54	Myristic acid	0.93	-6.40	0.11
Leupeptin	0.31	-0.18	0.64	LPE 16:0	0.88	-6.19	0.13
Vercuronium	0.24	0.08	0.71	Humantenine	0.68	-4.22	0.26
Tuberostemonine	0.09	0.02	0.88	Dodecylbenzenesulfonic acid	0.55	-4.03	0.37
				**24 h**			
Positive ionization				***Negative ionization***			
Alisol B Acetate	1.92	-2.49	< 0.01	Dehydroisoandrosterone sulfate	1.78	-5.88	0.08
Linoleic acid	1.76	-0.87	< 0.01	Sulfolithocholic acid	1.72	-5.80	0.09
3,6,9,12-Tetraoxatetracosan-1-ol	1.69	-1.04	< 0.01	Lichesterinic acid	1.70	-5.80	0.10
Adenine	1.63	-0.64	0.01	Rauwolscine	1.63	-5.55	0.12
Panaxatriol	1.59	0.84	0.02	DL-2-hydroxyvaleric acid	1.24	-4.14	0.26
Acetyl tributyl citrate	1.45	-0.64	0.04	Mitragynine	1.13	-3.72	0.31
Stearamide	1.39	0.89	0.05	2-Hydroxyisocaproic acid	1.06	-3.82	0.35
Dibutyl phthalate	1.40	-0.54	0.06	Lauric acid	0.97	-3.74	0.41
Vercuronium	1.30	0.51	0.08	Dodecanedioic acid	0.86	-3.74	0.45

Baclofen	1.28	0.37	0.09	L-lactate	0.85	-3.63	0.45
Anthranoyllycoctonine	1.11	-0.29	0.17	Stearic acid	0.85	-3.72	0.46
Erucamide	1.05	0.33	0.18	Undecanedioic acid	0.84	-3.61	0.46
Homoorientin	0.88	-0.36	0.26	Indoxyl sulfate	0.84	-3.71	0.46
4-Methylcoumarin	0.89	0.27	0.27	Azelaic acid	0.84	-3.62	0.46
Homoserine lactone	0.80	-0.30	0.31	Decanedioic acid	0.83	-3.64	0.46
Lauryl diethanolamide	0.72	0.19	0.38	Myristic acid	0.82	-3.88	0.47
N-lauroylethanolamine	0.63	-0.22	0.45	Thymol-β-D-glucoside	0.79	-3.68	0.48
Leupeptin	0.52	0.12	0.53	Benzenesulfonic acid	0.75	-3.55	0.51
Phthalic anhydride	0.51	-0.21	0.55	Trifluoroacetic acid	0.75	-3.49	0.51
Myristoyl ethanolamide	0.51	-0.20	0.55	Ortophosphate	0.73	-3.52	0.52
L-Pipecolic acid	0.45	-0.19	0.57	Methoxyfenozide	0.68	-3.49	0.55
Proline	0.45	-0.15	0.59	Lauryl sulfate	0.67	-3.58	0.56
Imidazole	0.41	-0.21	0.65	Zinniol	0.62	-3.39	0.58
Triphenylphosphate	0.23	-0.15	0.79	Humantenine	0.61	-3.45	0.59
Tuberostemonine	0.20	-0.13	0.82	Canrenone	0.61	-3.41	0.60
4-Methoxycinnamic acid	0.19	0.02	0.82	LPE 16:0	0.60	-3.14	0.60

Gamabufotalin	0.15	0.01	0.85	Dodecylbenzenesulfonic acid	0.60	-0.98	0.52
Diethanolamine	0.04	-0.06	0.94	Ascorbic acid	0.54	-3.57	0.64
Morpholine	0.04	-0.05	0.96	Allantoin	0.44	-3.52	0.70

Abbreviations: BA = barley group; BALA = 5% of lactic acid-treated barley group; VIP = variable importance in projection
