# Supplementary material for: Metabolomic and morphologic surveillance reveals the impact of lactic acid-treated barley on in vitro ruminal fermentation
Source: Anim Biosci. 2024 May 7;37(11):1901–12. doi: 10.5713/ab.23.0550 (PMC11541031; doi:10.5713/ab.23.0550)
Supplement: Supplementary file 2 [file ab-23-0550-Supplementary-Fig-1.pdf]

6 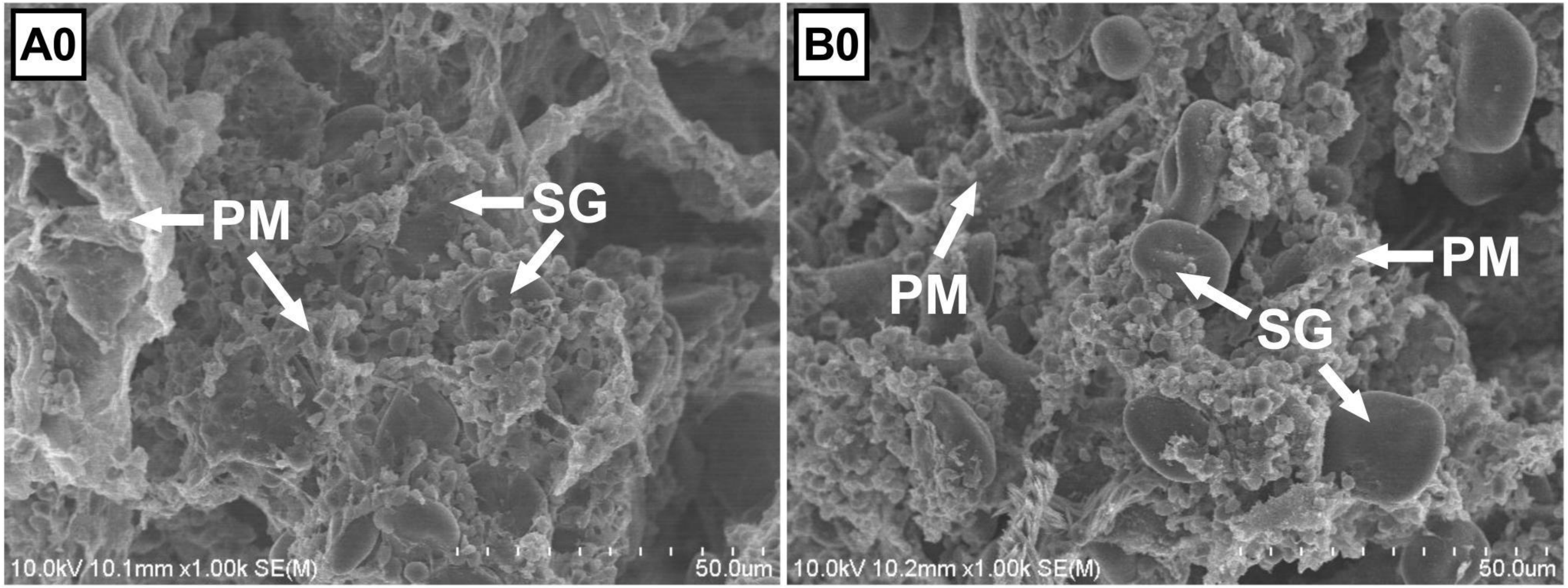
A0
PM
SG
10.0kV 10.1mm x1.00k SE(M)
50.0um
B0
PM
PM
SG
10.0kV 10.2mm x1.00k SE(M)
50.0um

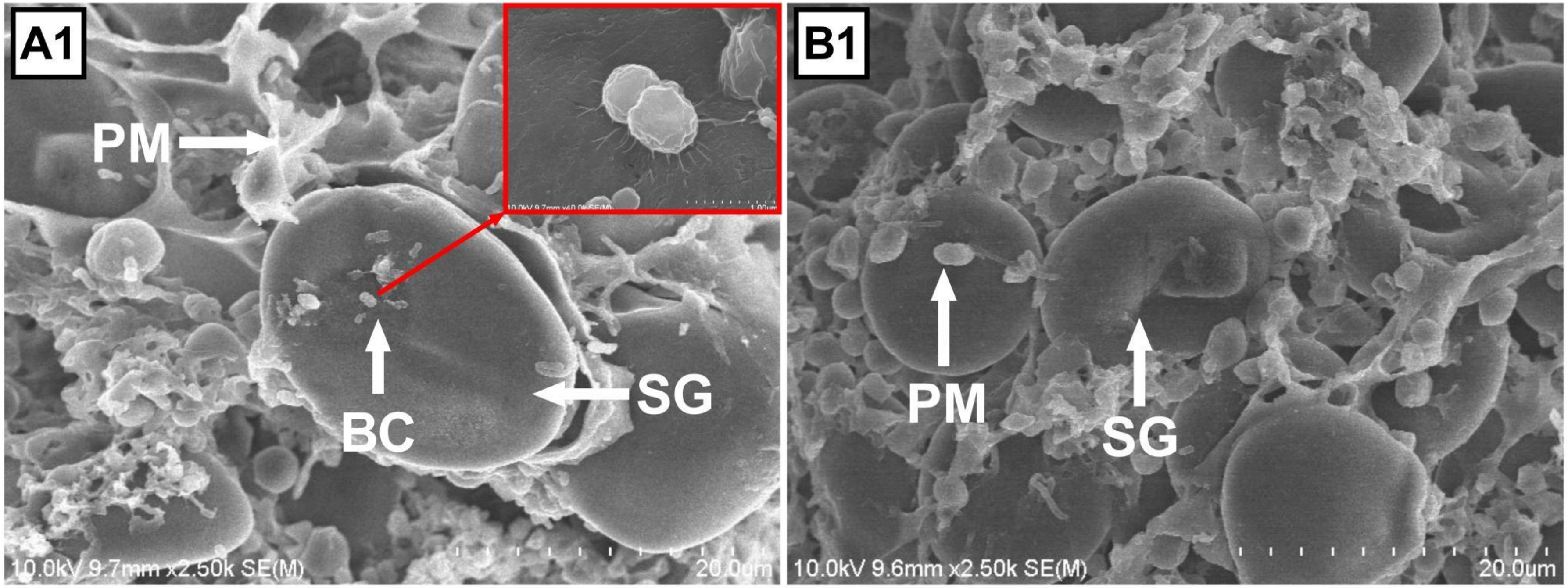
A1
PM
BC
SG
10.0kV 9.7mm x2.50k SE(M)
20.0um
B1
PM
SG
10.0kV 9.6mm x2.50k SE(M)
20.0um

8

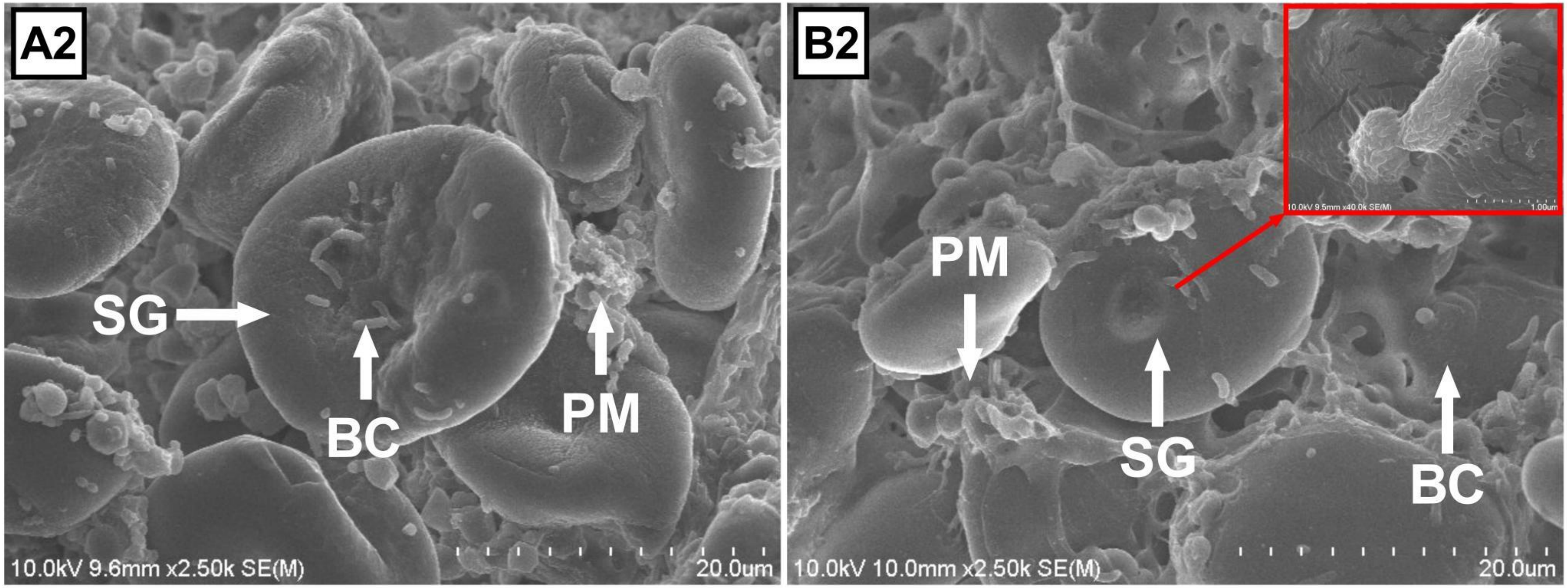
A2
SG
BC
PM
10.0kV 9.6mm x2.50k SE(M)
20.0um
B2
PM
SG
BC
10.0kV 10.0mm x2.50k SE(M)
20.0um

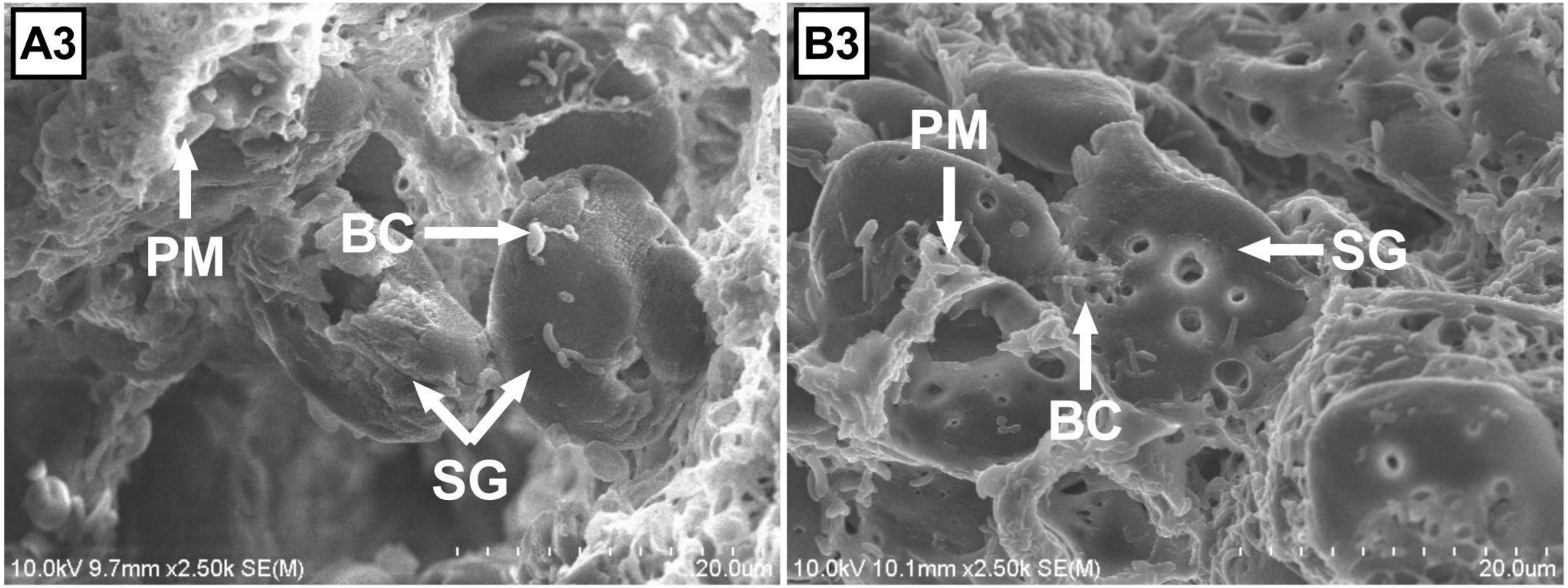
A3
PM
BC
SG
10.0kV 9.7mm x2.50k SE(M)
20.0um
B3
PM
SG
BC
10.0kV 10.1mm x2.50k SE(M)
20.0um

9

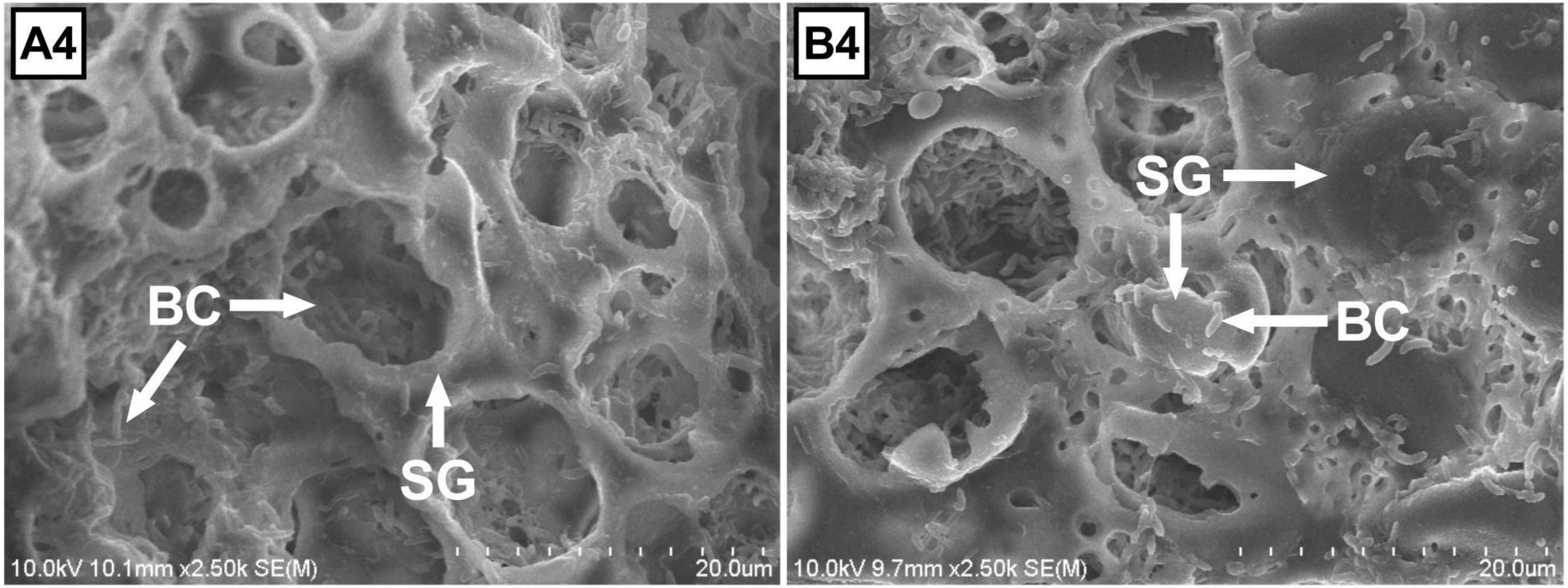
A4
BC
SG
10.0kV 10.1mm x2.50k SE(M)
20.0um
B4
SG
BC
10.0kV 9.7mm x2.50k SE(M)
20.0um

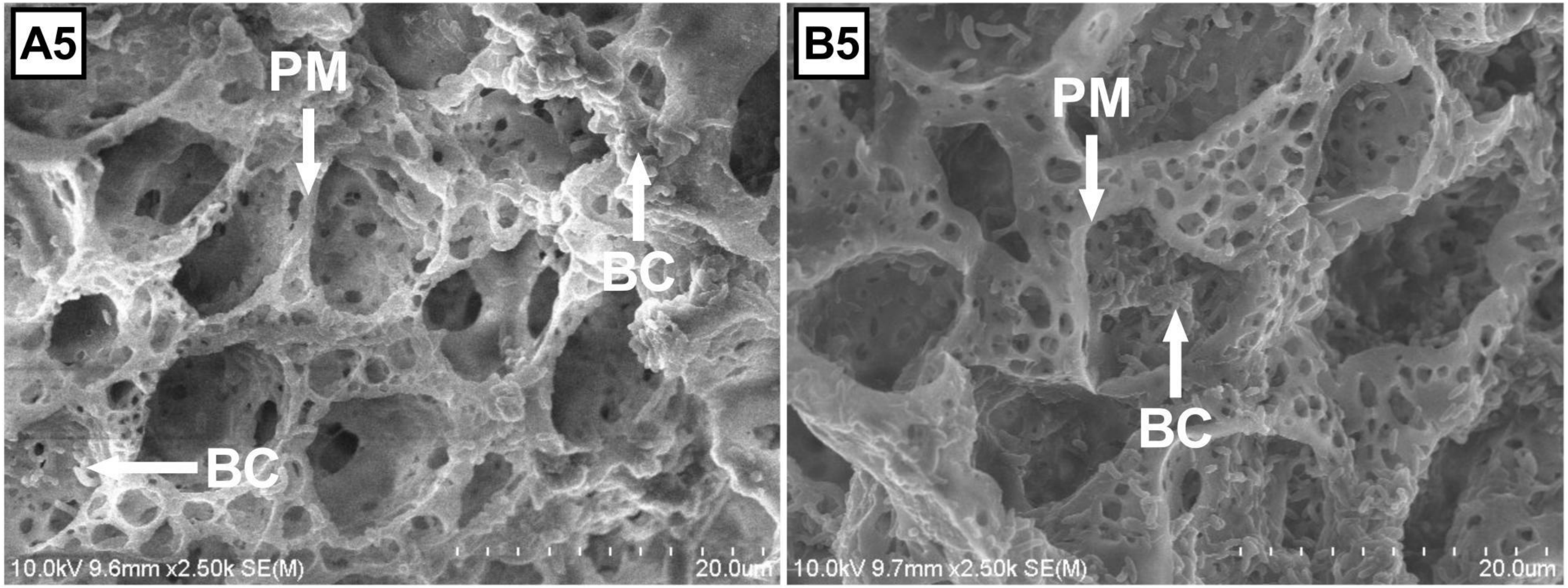

Supplementary Figure S1

Morphological features (2500×) of barley (A) and 5% lactic acid-treated barley (B) viewed by scanning electron microscopy at 0 (A0, B0), 3 (A1, B1), 6 (A2, B2), 12 (A3, B3), 18 (A4, B4), and 24 (A5, B5) h of fermentation. Bacterial cells began to appear on the SEM image at 3 h for BA but at 6 h for BALA. Larger images are shown in Supplementary Figure 1. BC, bacterial cells; SG, starch granule; PM, proteinaceous matrix.
